# The first report of the evaluation of the knowledge regarding toxoplasmosis among health professionals in public health centers in Rabat, Morocco

**DOI:** 10.1186/s41182-020-00208-9

**Published:** 2020-04-09

**Authors:** Majda Laboudi, Sanaa Ait Hamou, Imane Mansour, Ilham Hilmi, Abderrahim Sadak

**Affiliations:** 1 Department of parasitology, National Institute of Hygiene, Rabat, Morocco; 2 Faculty of Science Ben M’Sik, University Hassan II, Casablanca, Morocco; 3 Faculty of Science, University Mohamed V, Rabat, Morocco

**Keywords:** Toxoplasmosis, Health professionals; Knowledge, Rabat, Morocco

## Abstract

**Background:**

The assessment of the knowledge of *Toxoplasma gondii* infection among health professionals is essential to design an effective management strategy. The current study was conducted to assess the knowledge and perception of health professionals working in urban public health centers of different parts of Rabat in Morocco.

**Methods:**

A cross-sectional study was conducted from March 15 to June 15, 2017, in urban public health centers selected in the prefecture of Rabat in Morocco. A structured questionnaire was completed by participants and included questions on the epidemiology and diagnosis of toxoplasmosis and clinical issues related to the infection.

**Results:**

Ninety-six health professionals participated, including medical doctors, nurses, midwives, and laboratory technicians. Most of them were female (86, 89.58%). The mean age was 40.51 ± 10.26 years, and the mean length of time working in the field of healthcare was 15.92 ± 8.55 years. Eighty one percent (86, 81.25%) of health professionals knew the agent of toxoplasmosis, and 62 (64.5%) knew the definitive host of the parasite. Regarding clinical symptoms, 55 (57.29%) of the respondents knew that toxoplasmosis is an asymptomatic disease in immunocompetent persons. More than half of the respondents correctly identified the main routes of transmission: eating raw or undercooked meats, unwashed fruits and vegetables, and having direct contact with cats. However, only 29 (30.21%) of them believed that water can be a risk factor for the transmission of toxoplasmosis. Regarding diagnosis, only 14 (14.58%) health professionals knew about the avidity test.

**Conclusions:**

The implementation of educational interventions is recommended to raise awareness of toxoplasmosis among health professionals who provide prenatal care in public health centers.

## Background

Toxoplasmosis is a zoonotic parasitic disease caused by the intracellular protozoan *Toxoplasma gondii*. This parasite can infect humans and all warm-blooded animals, including mammals and birds [1]. According to the Food and Agriculture Organization and the World Health Organization, toxoplasmosis is ranked fourth among the 24 most harmful food-borne pathogens [2]. It has a widespread global distribution, as approximately 30% of the world’s population has chronic *T. gondii* infection [1]. Although it is typically a benign condition in an immunocompetent person, it is severe in immunocompromised patients and in cases of congenital infection. This latter is caused by the passage of the parasite from the mother to the fetus during pregnancy. *T*. *gondii* can infect the fetus with variable severity depending on the trimester of pregnancy and the effectiveness of the placental barrier. The risk of congenital infection is relatively low in the first trimester (10–15%) but is higher when the infection occurs during the third trimester (60–90%) [3]. However, clinical manifestations of congenital toxoplasmosis may be particularly severe when fetal contamination occurs during the first trimester of pregnancy. It can lead to severe fetopathy, such as hypotonia, microphthalmia, microcephaly, and chorioretinitis. The overall annual incidence of congenital toxoplasmosis in the world was estimated at 190,100 cases. This is equivalent to a burden of 1.20 million disability-adjusted life years (DALYs), which are the highest incidence rates occurring in South America, some countries in the Middle East, and other low-income countries [4].

Regarding the burden of congenital toxoplasmosis, the conference on toxoplasmosis held at the Centers for Disease Control and Prevention in 1998 recommended determining the knowledge and practices related to toxoplasmosis for obstetricians and gynecologists [5]. Therefore, updating knowledge about toxoplasmosis among health professionals (HPs) is of considerable importance in the prevention of congenital toxoplasmosis.

Several studies in many countries in the world have focused on assessing the knowledge and perception of toxoplasmosis among HPs [6–9]. However, in Morocco, there is a lack of information about the incidence of congenital toxoplasmosis. A recent review study found that the mean seroprevalence of toxoplasmosis is between 47 and 50% among pregnant women and 62% among immunocompromised persons [10], in addition to identifying the highest risk factors among pregnant women as contact with soil and lack of knowledge [11]. Hence, there is currently no report about awareness and knowledge concerning toxoplasmosis, its transmission routes, its clinical manifestations, its diagnosis, and attitudes toward prevention. Our study is the first to analyze the knowledge and awareness of toxoplasmosis in a sample among HPs at the level of urban health centers in the prefecture of Rabat in Morocco.

## Methodology

### Study area

**Fig. 1:**
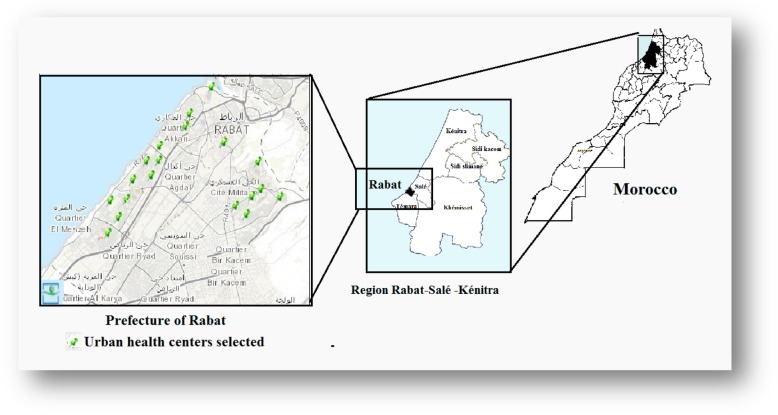
Map of Morocco, region of Rabat-Salé-Kénitra, prefecture of Rabat with urban health centers selected

The prefecture of Rabat is an administrative subdivision of the Moroccan region of Rabat-Salé-Kénitra (34° 02′ 00″ North, 6° 50′ 00″ West). On the administrative level, the prefecture of Rabat, which is made up of a part of the urban commune of Rabat, is divided into five districts: Hassan, Agdal-Ryad, el-Youssoufia, Yacoub el-Mansour, and Souissi districts (Fig. 1). Rabat is the capital of Morocco and has a Mediterranean climate, and the annual precipitation is 302.7 mm. The land area is 11,850 ha (118.5 km^2^), and the total population is 577,825 inhabitants (in 2014).

### Study design and sample subjects

The urban health center is the first contact urban health facility. It implements therapeutic patient education, prevention, and public health activities, including the monitoring of the health of mothers and children. This cross-sectional study was carried out among health professionals (HPs) practicing in public health centers selected in the prefecture of Rabat in Morocco from March 15 to June 15, 2017. The HPs include medical doctors (general practitioners, pediatricians, and gynecologist doctors), nurses (versatile nurses), midwives, and laboratory technicians. Given the inability to identify all the HPs working at urban health centers in the prefecture of Rabat, the recruitment of our study population will be based on convenience sampling. The number of participants from public urban health centers was selected according to voluntary participation in the study. The objective of the study was explained to the HPs before they were asked to complete the questionnaire. Questionnaires that were not completed in full were considered invalid; therefore, they were excluded.

### Questionnaire

The data were collected using a questionnaire focusing on knowledge of toxoplasmosis among HPs. The questionnaire was composed of four sections: demographic characteristics of the population (5 items), epidemiology knowledge (6 items), clinical manifestations (6 items), and diagnosis and treatment knowledge related to toxoplasmosis (6 items). The questionnaire was anonymous and self-administered and was filed in the workplaces of the HPs.

### Statistical analysis

All the data from the questionnaires were statistically analyzed using the EpiInfo software (version 3.5.4 (CDC, USA, 2012)). Descriptive statistics are presented in tabular format.

## Results

### Demographic characteristics of health professionals

A total of 170 questionnaires were distributed to HPs, of which 120 (70.58%) were returned. Approximately 25% of the returned questionnaires were incomplete and were therefore excluded, leaving a total of 96 valid questionnaires for statistical analysis.

Of the 96 HPs participating in the present study, 54 (56.25%) were medical doctors, 19 (19.79%) were nurses, 14 (14.58%) were midwives, and 9 (9.38%) were laboratory technicians. The mean age of the HP participants was 40.51 ± 10.26 years (range 22 to 58 years); furthermore, most HPs were female (86, 89.58%), while 10 (10.42%) were male. The majority of the participants (29, 33.72%) were older than 45 years old, while 12 (13.95%) were in the 31- to 35-year-old age group. The mean length of time working in the field of healthcare was 15.92 ± 8.55 years (range 1 to 38 years) (Table 1). Regarding education level, 46 (47.92%) had studied between 4 and 7 years after baccalaureate, and 8 (8.33%) had more than 7 years of study after baccalaureate (Table 1).

**Table 1:** Demographic data of the population studied

Characteristic	No. of HPs	%
**Gender**		
Female	86	89.58
Male	10	10.42
**Age ( years old ), mean 40.51 ± 10.26** years		
≤ 30	18	20.93
31–35	12	13.95
36–45	27	31.40
> 45	29	33.72
**Time from graduation after baccalaureate**		
≤ 3 years	42	43.75
4–7 years	46	47.92
> 7 years	8	8.33
**Length of service years in public health (years), mean 15.92 ± 8.55 years**		
≤ 10	23	27.06
11–15	20	23.53
> 15	42	49.41
**Type of professional work**		
Medical doctors	54	56.25
Midwives	19	19.79
Nurses	14	14.58
Laboratory technicians	9	9.38

### Knowledge of the parasite and the epidemiology of toxoplasmosis

Most participants correctly answered that toxoplasmosis is parasite infection caused by protozoa *T*. *gondii* (78 participants, 81.25%) having felids as definitive hosts (62 participants, 64.58%)*.* More than half of the respondents correctly identified the routes of transmission: eating raw or undercooked meats, unwashed fruits and vegetables, and having direct contact with cats. However, less than half (29, 30.21%) of the respondents correctly responded that untreated water can be a risk factor for the transmission of toxoplasmosis (Table 2).

**Table 2:** Knowledge of parasite epidemiology, clinical aspects, diagnosis, and treatment of toxoplasmosis

Questions and answers	Frequency	%
**Epidemiology**		
**Do you know the agent of the toxoplasmosis is*Toxoplasma gondii***		
Yes*	78	(81.25)
No	5	(5.21)
Do not know	13	(13.54)
**Do you know the definitive host of toxoplasmosis**		
Yes*	62	(64.58)
No	34	(35.42)
**Infection with*T. gondii*can be acquired by**		
**Eating undercooked meat**		
Yes*	87	(90.63)
No	2	(2.08)
Do not know	7	(7.29)
**Drinking untreated water**		
Yes*	29	(30.21)
No	50	(52.08)
Do not know	17	(17.71)
**Direct contact with cat**		
Yes*	78	(81.25)
No	5	(5.21)
Do not know	13	(13.54)
**Fruits and vegetables having contact with cat stool**		
Yes*	79	(82.29)
No	6	(6.25)
Do not know	11	(11.46)
**Clinical aspects**		
**Do you know that the most cases of toxoplasmosis in immunocompetent person are asymptomatic**		
Yes*	55	(57.29)
No	18	(18.75)
Do not know	23	(23.96)
**Do you know that the seroconversion among pregnant women is defined when**		
Negative serology become positive during pregnancy*	72	(75.00)
Positive serology become negative during pregnancy	7	(7.29)
Do not know	17	(17.71)
**Do you know that the congenital toxoplasmosis defined when**		
Passage of parasite from mother to fetus*	46	(47.92)
In case of seroconversion during pregnancy	32	(33.33)
Do not know	18	(18.75)
**Do you know that the highest-risk period of toxoplasmosis transmission can be acquired in the 3rd trimester**		
Yes*	15	(15.63)
No	63	(65.63)
Do not know	18	(18.75)
**Do you know that the highest period of severity of lesion can be acquired in 1st trimester**		
Yes*	36	(37.50)
No	37	(38.54)
Do not know	23	(23.96)
**Do you know that toxoplasmosis is an opportunist disease in immunocompromised person**		
Yes*	36	(37.50)
No	45	(46.88)
Do not know	15	(15.63)
**Diagnosis and treatment**		
**Do you know that the antenatal screening for*T. gondii*infection based on*antitoxoplasma*-specific IgG and IgM detection**		
Yes*	71	(73.96)
No	9	(9.38)
Do not know	16	(16.67)
**Do you know the anti-toxoplasma IgG avidity test**		
Yes*	14	(14.58)
No	82	(85.42)
**Do you know the diagnostics of congenital toxoplasmosis in utero**		
Yes*	38	(39.58)
No	58	(60.42)
**Do you know the treatment in case of seroconversion to prevent the transmission of the parasite to the fetus**		
Yes*	43	(44.79)
No	53	(55.21)
**Do you know that the prophylaxis of toxoplasmosis must be recommended among seronegative pregnant women**		
Yes*	70	(72.92)
No	11	(11.46)
Do not know	15	(15.63)
**Does the human vaccine of toxoplasmosis exist**		
Yes	20	(20.83)
No*	57	(59.38)
Do not know	19	(19.79)

*The correct answer

### Knowledge of the clinical aspects, diagnosis, and treatment of toxoplasmosis

Approximately 55 (57.29%) HPs knew that toxoplasmosis is asymptomatic in most cases. Most HPs (72, 75%) knew the exact definition of seroconversion, and only 46 (47.92%) knew the definition of congenital toxoplasmosis (Table 2).

Few HPs (15, 15.63%) knew the highest-risk period of transmission of the parasite during pregnancy, and only 36 (37.50%) knew about the highest period of gravity of the lesion that can affect the child*.* On the other hand, less than half (36 participants, 37.50%) knew to categorize toxoplasmosis as an opportunistic disease in an immunocompromised person due the reactivation of the parasite.

Nearly 74% of participants (71 participants, 73.96%) knew that the serological diagnosis of toxoplasmosis was realized by ELISA based on the detection of IgG and IgM *T. gondii* antibodies, and only 14 (14.58%) of HPs knew that the avidity test can be applied as a complementary serology technique to date the primary infection during the first months of pregnancy (Table 2).

Fewer HPs (38, 39.58%) answered that they gained only a very limited amount of knowledge about diagnosis in utero.

For treatment, less than half of the HPs (43, 44.79%) knew the proper toxoplasmosis medication for pregnant women in case of seroconversion to prevent the transmission of the parasite to the fetus, during pregnancy. Furthermore, 57 HPs (59.38%) reported the absence of a human vaccine for toxoplasmosis, while 19 (19.79%) were unaware of the lack of a vaccine (Table 2).

## Discussion

In Morocco, healthcare facilities represent the first line of resort for patients by providing curative and preventive healthcare services and undertaking health promotion activities [12]. Therefore, it is necessary for HPs to have accurate information on various health problems in general and pertaining to maternal health in particular, such as toxoplasmosis, so that they can become involved in prevention and control activities. In this framework, we surveyed HPs working in health facilities in the prefecture of Rabat in Morocco to determine their knowledge, prevention, and treatment practices for *T*. *gondii* infection.

In the current study, most of the HPs correctly answered questions related to knowledge of the infectious agent (over 80%) and the definitive hosts (64.58%). These results are consistent with those of a study conducted in Mexico, where most participants correctly answered that *T*. *gondii* is a parasite (89.6%) and that cats are definitive hosts (83.8%) [13].

Regarding knowledge of risk factors, the majority of HPs recognized the main route of contamination through the consumption of undercooked meat or fruits and vegetables containing tissue cysts and direct contact with cat feces. However, only 30.21% of HPs knew that untreated water could transmit the toxoplasmosis parasite. Several publications in many countries in the world highlighted similar results. In Ethiopia, Abebe et al. reported that only 6.1% of study participants mentioned that drinking unboiled water is an important mode of transmission [8]. In Nigeria, less than one-third (28%) of the medical doctors participating in the study knew that the *T*. *gondii* infection can be a waterborne disease [9], and in Mexico, only slightly higher than one-third of respondents knew that water can transmit the toxoplasmosis parasite (13%) [13]*.*

Many global studies have reported evidence of waterborne parasites. In France, Velenna et al. reported the presence of *T*. *gondii* oocysts in 7% of raw surface-water samples [14]. In addition, *T*. *gondii* oocysts were responsible for waterborne outbreaks between January 2004 and December 2010 in Mexico [15]. In Brazil, waterborne *T*. *gondii* was thought to be responsible for an outbreak involving 155 persons served by an underground tank reservoir delivering unfiltered water [16]. Furthermore, the presence of *T*. *gondii* in public drinking water was reported in Mexico*.* Toxoplasmosis can be acquired through the ingestion of contaminated drinking water with oocysts of *T*. *gondii*, which are highly resistant to the routine chlorination disinfection processes that are commonly used in the water supply industry [17]*.*

The current study findings indicate that 75% of HPs knew the definition of seroconversion, while 47.92% knew the definition of congenital toxoplasmosis. These findings probably reveal that the HPs seem to have thought that following the contraction of the parasite by pregnant women, there is systematically congenital toxoplasmosis. Indeed, seroconversion manifests when pregnant women develop IgG antibodies against *T*. *gondii* during pregnancy, with a significant increase in IgG antibody titers in the presence of IgM antibodies. However, congenital toxoplasmosis confirmed the passage of the parasite from mother to child as a transmitted disease during pregnancy [18].

Remarkably, unexpected results were reported among HPs about the period of risk during pregnancy; only 15.63% of HPs knew about the highest-risk period of transmission of the parasite during pregnancy, whereas 37.5% knew about the highest-risk period of the gravity of the lesion for the child*.* The erroneous knowledge observed in our finding regarding the highest risk of congenital infection in the first trimester of pregnancy has also been reported among obstetricians and gynecologists in Mexico [7] and the USA [6]. The risk of congenital infection is relatively lower during the first trimester (10–15%) and highest when the infection occurs during the third trimester (60–90%). However, congenital infection during the first trimester can lead to more severe disease when it occurs [3].

Nearly 57.29% of HPs reported that toxoplasmosis is asymptomatic in most cases. Previous studies carried out in many countries indicated that the majority of the knowledge of *T*. *gondii* infection is mostly subclinical: 73.9% in Brazil [19], 59.0% in Mexico [7], and 69.92% in Nigeria [9]. On the other hand, less than half knew that toxoplasmosis is an opportunistic disease in immunocompromised persons (37.5%) due to the reactivation of the parasite. Our results yielded fewer accurate responses than those in Ethiopia, where 52.7% of HPs identified toxoplasmosis as an important pathogen in HIV-infected patients and pregnant women [8]. Toxoplasmosis has emerged as a major opportunistic disease in patients with acquired immunodeficiency syndrome (AIDS). It can manifest as potentially fatal encephalitis due to the reactivation of latent infections in HIV-associated immune suppression [20]. Toxoplasmosis is also a clinically important opportunistic infection in other immunosuppressed individuals, such as patients who have had an organ transplant or who are undergoing cancer treatment [21]*.*

According to the knowledge of the diagnosis, the vast majority (over 70%) of HPs knew about the detection of anti-*T*. *gondii* IgG and IgM antibodies as the pathway test for the diagnosis of toxoplasmosis, while 82 (85.42%) did not know about the avidity test as a complementary technique used in serology as a dating test for infection during the first months of pregnancy. Similarly, the lack of knowledge of the avidity test found in the present study has also been reported by physicians caring for pregnant women in Mexico and the USA, where 90.1% and 87.3% of HPs, respectively, did not know about the avidity test [13, 22].

Indeed, IgM detection was not always considered a sufficient argument to conclude recent infection [23]. Therefore, the avidity test seems to be effective and reliable, allowing the exclusion of evolutionary toxoplasmosis [24–26]. A study carried out in the Rabat region in Morocco showed that the avidity test of anti-*T*. *gondii* IgG made it possible to exclude seroconversion of less than 20 weeks in 72.2% of cases [27]. However, polymerase chain reaction-based molecular techniques are also useful for the diagnosis of *T*. *gondii* infection in cases of proven fetal infection (positive amniocentesis) or strongly suspected late maternal contamination [13]. It is generally known that cases of serological suspicion of primary infection should be confirmed with additional tests, such as the avidity-IgG test [7]. The use of PCR tests for the confirmation of *T. gondii* infection or molecular tests to detect *T. gondii* DNA is very rare in Morocco, which could explain the low knowledge (39.58%) reported in the current study about the diagnosis of congenital toxoplasmosis in utero.

With regard to treatment knowledge, less than half of HPs (44.79%) knew the proper medication for toxoplasmosis in pregnant women in cases of seroconversion to prevent the transmission of the parasite to the fetus. Nevertheless, 55.21% of respondents did not know which treatment should be administered if the pregnant woman becomes *T*. *gondii* seropositive during pregnancy. In Mexico, most doctors had incomplete knowledge about the treatment of congenital toxoplasmosis; only 23% of doctors knew what medications are recommended against congenital toxoplasmosis in pregnant women. Another study undertaken in Mexico updated the knowledge of toxoplasmosis among HPs and reported that up to 55.7% of participants provided incorrect answers regarding the interpretation of serology tests for the treatment of pregnant women [13].

The primary prevention measures for the treatment of toxoplasmosis of the fetus are based on taking spiramycin (Rovamycin®), which should reduce the transplacental passage of the parasite, but its effectiveness has not been demonstrated. However, secondary prevention, namely, pyrimethamine-sulfadiazine and folinic acid, is prescribed to reduce the risk and severity of sequelae when fetal contamination has been proven (positive amniocentesis) or is strongly suspected (late maternal contamination). Although the efficacy of the treatment pre- and postnatally has not been clearly demonstrated, many arguments are in favor of a reduction in the severity of sequelae in treated individuals [18].

Furthermore, nearly 60% of HPs reported the absence of the human vaccine for toxoplasmosis. Indeed, there are no human vaccines against toxoplasmosis. In the absence of an effective vaccine in humans, following the best preventive practice remains the best way to approach the problem of toxoplasmosis; this must be done by limiting exposure to oocysts or tissue cysts [18].

## Limitations of the study

The sample size was considered the main limitation of this study due to the refusal of HPs to participate directly or by absence during the time of data collection. However, this is the first report on knowledge of toxoplasmosis among HPs in Morocco; it will be useful for the design of strategies leading to optimal education about *T. gondii* infection. The use of other studies with a larger sample size would help to assess the actual knowledge levels of HPs.

## Conclusions

This study revealed that HPs practicing at urban health centers in Rabat in Morocco have high levels of knowledge related to the epidemiology of toxoplasmosis and low levels of knowledge about untreated water as a risk factor for the transmission of the parasite disease. In addition, moderate knowledge among HPs was reported regarding the clinical diagnosis of toxoplasmosis. Hence, the information derived from this study allows us to make recommendations regarding the sensitization of the doctors and nurses on diagnosis, treatment, and clinical of toxoplasmosis throughout workshops, lectures, and other training classes arranged by the Moroccan Ministry of Health to update the knowledge of toxoplasmosis among HPs. Further studies are required to evaluate the knowledge, practice, and attitude of HPs on toxoplasmosis with a large sample size in the same study area.

## Data Availability

The data supporting the conclusions of this article are included in the article

## Abbreviations

HPs: Health professionals
*T*. *gondii*: *Toxoplasma gondii*
